# Study on the relationship between hyperthyroidism and vascular endothelial cell damage

**DOI:** 10.1038/s41598-020-62796-0

**Published:** 2020-04-24

**Authors:** Tianlong Yu, Miao Jing, Yunyan Gao, Chang Liu, Lanchun Liu, Haihan Jia, Peng Liu, Manli Chang

**Affiliations:** 1 Key lab of Etiology and Epidemiology, Education Bureau of Heilongjiang Province & Ministry of Health, Microelement and human health lab of Heilongjiang Province, Center for Endemic Disease Control, Chinese Center for Disease Control and Prevention, Harbin Medical University, Harbin, 150081 China; 2 Harbin Center of Disease Control and Prevention, Harbin, 150081 China; 3 The Second Affiliated Hospital of Harbin Medical University, Harbin, 150081 China

**Keywords:** Cerebrovascular disorders, Thyroid diseases

## Abstract

The aim of the research is to explore the relationship between hyperthyroidism, iodine, antithyroid drugs (propylthiouracil) and vascular endothelial injury. In total, 136 SD rats were randomly allocated into the control group, the hyperthyroidism group, the hyperthyroidism propylthiouracil group, the hyperthyroidism low iodine group, the high iodine group, and the endothelial injury group. Rats were raised for 60 days. Afterward, indicators concerning endothelial damage were determined, including the von Willebrand Factor (vWF), thrombomodulin (TM), nitric oxide (NO), endothelin 1 (ET-1), and P-selectin, as well as the plant hemagglutinin sample type oxidized low-density lipoprotein receptor 1 (LOX-1) from the aorta and the number of endothelial progenitor cells (EPCs) in whole blood. The hyperthyroidism group had significantly higher values for vWF, TM, NO, ET-1, and P-selectin in serum and a higher number of EPCs in whole blood compared with the control group, similar to the LOX-1 expression in abdominal aorta. The hyperthyroidism low iodine group had significantly higher values for vWF, ET-1, and P-selectin in serum and a higher number of EPCs in whole blood compared with those of the control group, as was the case for LOX-1 expression in the abdominal aorta. The hyperthyroidism propylthiouracil group had significantly higher values for FT_4_ in the serum compared with those in the control group. The electron microscope showed that hyperthyroidism caused a certain degree of endothelial injury to the abdominal aorta in rats. Hyperthyroidism can damage the vascular endothelium and is a high-risk factor for cardio-cerebrovascular disease. Propylthiouracil could be used in the treatment of hyperthyroidism, thus protecting endothelial cells from damage.

## Introduction

Hyperthyroidism is caused by the synthesis and release of excessive thyroid hormones. Hyperthyroidism is a serious endocrine disease in the population and includes symptoms such as heart palpitations, sweating, increased appetite, diarrhea and weight loss. Most patients experience exophthalmos, eyelid edema, vision loss and other symptoms^1^. Patients with hyperthyroidism usually have faster heart rates and strong metabolism; when these symptoms are left untreated, they might give way to vascular endothelial dysfunction and eventual damage to vascular endothelial cells, according to previous research^2^. The potential effect of hyperthyroidism on the development of rat adrenal glands is mediated by vascular endothelial growth factor (VEGF) expression, angiogenesis, and apoptosis^3^. When treating hyperthyroidism, it is usually required that patients reduce their iodine intake, and antithyroid drugs such as propylthiouracil (PTU) are administered. This treatment might also damage endothelial cells as the thyroid hormone changes due to the reduced intake of iodine and the use of PTU to treat hyperthyroidism, which might induce anti-neutrophil cytoplasmic antibody (ANCA)-related vasculitis, can cause some damage to the endothelial cells. Moreover, research has shown that a high iodine intake can lead to endothelial cell activation injury and hyperplasia, thereby increasing subendothelial extracellular matrix, promoting intimal thickening, thickening the inferior vena cava wall, and causing stenosis; there is a dose-response relationship between iodine and endothelial cell activation injury^4^. Excess iodine exposure can increase oxidative stress, cause damage to vascular endothelial cells, and alter the expression of adhesion factors and the activity of nitric oxide synthase (NOS). These changes may explain the mechanisms underlying excess iodine-induced vascular injury^5^. Although the aforementioned list of potential causes of blood vessel damage is plausible, little evidence is found to support these statements. Additionally, the small number of references found that relate to this content are not very concrete or methodical. Based on the abovementioned limited evidence, this project simulated the pathogenesis and treatment process of PTU in the population and studied the relationship between hyperthyroidism, iodine, antithyroid drugs (propylthiouracil) and vascular endothelial injury by conducting an experiment with animals. This study explores the relationship between endothelial vascular damage and subsequent hyperthyroidism, and this study examined low iodine intake after hyperthyroidism, PTU treatment after hyperthyroidism, and high iodine intake as potential factors.

## Materials and Methods

### Animals and diets

Clean-grade SD rats (SD rats are a rat strain; SD rats were bred from Wistar rats on the Sprague-Dawley farm in the United States in 1925 and are widely used in pharmacology, toxicology, efficacy and GLP experiments) were used as experimental animals (six weeks old) from the animal center of Kunming Medical University. The rats were divided into six groups according to weight: (1) control group, with normal diet and deionized water (50 μg/L iodine added), feeding for 60 days; (2) hyperthyroidism model group, with thyroxine tablets (25 mg/100 g, Shanghai Changcheng Pharmaceutical Co., Ltd.) continuous gavage for 30 days (once daily), the main ingredients of this product are made from the thyroid gland of food animals such as pigs, cattle, and sheep. Thyroid hormones include thyroxine (T4) and triiodothyronine (T3); after successful establishment of the model, thyroxine was stopped, and rats were supplied with normal diet and deionized water (50 μg/L iodine added, model group) every day; (3) hyperthyroidism PTU group, beginning with continuous gavage of thyroxine tablets (25 mg/100 g) for 30 days (once daily); after successful establishment of the model, thyroxine was stopped, and the rats were supplied with normal diet and deionized water (50 μg/L iodine added), followed by a daily dose of 5 mg/kg PTU gavage (once daily) for 30 days (H + PTU group); (4) hyperthyroidism low iodine group, thyroxine tablets (25 mg/100 g), continuous gavage for 30 days (once daily). Thyroid hormones include thyroxine (T4) and triiodothyronine (T3); after successful establishment of the model, thyroxine was stopped, and the rats were supplied with normal diet and drinking deionized water (H + low iodine group) every day; (5) high iodine group, with normal diet and deionized water (3000 μg/L iodine); and (6) endothelial injury group, with normal diet and epinephrine hydrochloride (33.3 μg/100 g) injected subcutaneously five days before sacrifice, three times a day for five days. A total of 136 rats, including 80 females and 56 males, were involved. The control and hyperthyroidism model group had 28 rats, and the other groups (H + PTU group, H + low iodine group, high iodine group and the endothelial injury group) each had 20 rats. The proportion of female rats to male rats was 6:4 in each group (hyperthyroidism occurs more frequently in females than in males, and this proportion was proposed considering the sex ratio of patients in the hospital).

### Diet

The rat feed was purchased from Beijing Keaoxieli Feed Co., Ltd. For the water formula, potassium iodate was added to deionized water to prepare the iodine-containing deionized water with iodine concentrations of 50 μg/L and 3000 μg/L.

### Detection indicators and methods

Urine of SD rats was collected for 24 hours at  two months and stored in a refrigerator at −20 °C. Rats were weighed and then anesthetized with 0.3 ml of 10% chloral hydrate per 100 g of body weight. The rats were sacrificed, and the abdominal aorta was taken. Blood and whole blood were collected in EDTA anticoagulant tubes. Blood for serum separation was packed in a non-anticoagulated tube. After the blood was allowed to stand for  one hour, it was centrifuged at 3500 r/min for 15 min. The serum was separated and stored in a refrigerator at −80 °C. The thyroid gland and heart were weighed, and the 2-cm-long abdominal aorta was taken and divided into two parts: one stored in a nonenzyme cryotube and the other in 4% glutaraldehyde.

The body weight, thyroid weight, heart weight, and ratios of heart-body (h-body) and thyroid-body (T-body) were determined. For the determination of iodine in rat urine, the AS^3+^-Ce^4+^ catalytic spectrophotometry method was used (WS/T 107-2006). The content of free triiodothyronine (FT_3_) and free thyroxine (FT_4_) and the level of thyroid-stimulating hormone (TSH) in rat serum were determined by radioimmunoassay. In addition, the contents of the von Willebrand Factor (vWF), thrombomodulin (TM), nitric oxide (NO), endothelin 1 (ET-1) and P-selectin in rat serum were detected by ELISA kits (Beijing Bio topped Science Technology Co. Ltd.). The expression of the plant hemagglutinin sample type oxidized low-density lipoprotein receptor 1 (LOX-1) in the descending aorta was detected by real-time fluorescence quantitative PCR. After phenol-chloroform RNA extraction from the descending aorta, for purity monitoring with Micro spectrophotometer k2800 (Beijing Kaiao) and electrophoresis detection, cDNA (0.5 μl), the primer sequences for endogenous controls were R-GAPDH-F AGACAGCCGCATCTTCTTGT and R-GAPDH-R TGATGGCAACAATG TCCACT. The upstream primer is GGCCATCCTTTGCCTAT GT (0.5 μl), the downstream primer is ACATCTGCCCCTCCAGGATA (0.5 μl), SYBR® Premix Ex Taq™ (Tli RNaseH Plus) (2×) (10 μl) and dH_2_O (4.0 μl), finally using real-time PCR (Chromo4, Bio-Rad, USA), 95 °C, 30 s; 95 °C, 3 s; 60 °C, 34 s (Collect fluorescent signal); 45 cycles. For analysis of the melting curve, the temperature was 60 °C–95 °C, and the result was read every minute. The formula used for calculating the LOX-1 mRNA level is 2^−ΔΔCт^. The number of endothelial progenitor cells (EPCs) was detected by flow cytometry counting. Fluorescent antibody-labeled single cell suspensions were counted by flow cytometry and generally stopped after 5 minutes of collection of single cell suspensions. For computer software analysis, we first set up a single nuclear cell area in the scatter plot to exclude platelets, cell debris, doublet cells, microparticles, etc. A second door was set according to CD34-PE/SSC. The CD34-PE positive cell population region was delineated, and the third gate was used to select CD34-PE and CD133-FITC double positive cells. Finally, the CD34/CD133 (Bioss, Beijing) double fluorescent marker positive cells were counted. The Beckman MPL500 software was used to analyze the data. The processing of samples, reagents and the experimental environment were maintained at 20 ± 2 °C.

### Electron microscopy investigation

Electron microscopy was used to observe the ultrastructure of the descending aortic vascular endothelium. The steps included fixation, dehydration, infiltration and embedding, trimming and sectioning, and staining.

### Ethics committee approval

All methods were carried out in accordance with the guidelines for the use of experimental animals by Harbin Medical University, and all experimental protocols were approved by the Ethics Committee of Harbin Medical University (No. HMUe12.n5).

### Statistical analysis

Data were analyzed using SPSS® version 20.0 statistical software (IBM International Business Machines Corporation, New York). The urine iodine resulted in a skewed distribution and is expressed as the median, and normally distributed data are reported as the means ± SD. Normally distributed values were compared using one-way ANOVA, and further post hoc comparisons were conducted using SNK (multiple range tests). A P-value <0.05 was regarded as statistically significant.

## Results

All results were compared between each group and the control group. Additionally, the hyperthyroidism with low iodine group and the hyperthyroidism with PTU group were compared with the hyperthyroidism group; however, no significant differences were found.

### Hyperthyroidism modeling

During pre-experimentation, the thyroid function of rats in the control group and the hyperthyroidism model group were tested after 30 days of feeding (eight rats per group). The results indicated that the level of TSH in the hyperthyroidism model group (0.23 ± 0.09 uIU/ml) was lower than the level in the control group (1.58 ± 0.30 uIU/ml, P < 0.05). The FT_3_ and FT_4_ values in the hyperthyroidism model group (3.41 ± 2.82 pg/ml, 9.01 ± 2.12 ng/dl) were significantly higher compared with those in the control group (0.29 ± 0.14 pg/ml, 5.20 ± 0.84 ng/dl, P < 0.05). These results verified the success of the hyperthyroidism model when combined with symptoms of hyperthyroidism such as irritability and rapid breathing.

### Urinary iodine, heart-body ratio, thyroid-body ratio, and thyroid function among the six groups of SD rats

The median urinary iodine (MUI) of rats in the H + low iodine group (24.78 µg/L) was significantly lower than that in the control group (78.36 µg/L, P < 0.05), and the MUI of the high iodine group (2416.39 µg/L) was significantly higher than that of the control group (P < 0.05) (Table 1).

**Table 1:** Determination results of six groups of SD rats’ urinary iodine, heart - body ratio, thyroid - body ratio, and thyroid function.

Group	N	MUI (µg/L)	Thyroid-body ratio	heart-body ratio	TSH uIU/ml	FT_3_ pg/ml	FT_4_ ng/dl
Control	18	78.36	0.0044 ± 0.0009	0.3147 ± 0.0318	1.56 ± 0.44	0.95 ± 0.32	4.14 ± 0.72
Model	18	75.58	0.0062 ± 0.0020*	0.4183 ± 0.0630*	0.56 ± 0.24*	2.71 ± 0.6*	7.80 ± 1.50*
H + PTU	18	70.77	0.0051 ± 0.0017	0.3617 ± 0.0467*	1.71 ± 0.29	0.70 ± 0.25	5.07 ± 1.80*
H + low iodine	18	24.78*	0.0044 ± 0.0008	0.4393 ± 0.5900*	0.56 ± 016*	2.60 ± 0.72*	8.13 ± 1.13*
High iodine	18	2416.39*	0.0051 ± 0.0011	0.3366 ± 0.0600	1.33 ± 0.63	0.92 ± 0.35	4.37 ± 0.80
Endothelium injury	18	74.58	0.0046 ± 0.0014	0.3221 ± 0.0528	1.78 ± 0.46*	0.74 ± 0.35	5.24 ± 0.87

*Denotes comparison with control group, P < 0.05, MUI, median of urinary iodine, FT_3_, triiodothyronine, FT_4,_ thyroxine. H + PTU group, hyperthyroidism propylthiouracil group, H + low iodine, hyperthyroidism low iodine group.

For the thyroid-body ratio, when compared with the control group (0.0044 ± 0.0009), the ratio of the hyperthyroidism model group was higher (0.0062 ± 0.002, P < 0.05). For heart-body ratio, when compared with the control group, the ratios in the hyperthyroidism model group, H + PTU group and H + low iodine group were all higher (0.4183 ± 0.063, 0.3617 ± 0.0467, and 0.4393 ± 0.59, respectively; P < 0.05; Table 1). For thyroid function, compared with the control group, the hyperthyroidism model group had high TSH levels, and the H + low iodine group had lower levels of TSH (P < 0.05), while the endothelium injury group had higher serum levels of TSH (P < 0.05). Compared with the control group, the hyperthyroidism model group, the H + PTU group, and the H + low iodine group had significantly higher FT_4_ (P < 0.05), as shown in Table 1.

### Endothelial injury results

Compared with the control group (13.17 ± 2.69 µmol/L), the content of NO in the hyperthyroidism model group (25.40 ± 7.72 µmol/L) and the endothelial injury group (32.60 ± 8.90 µmol/L) was significantly higher (P < 0.05). Compared with the control group (12.99 ± 1.11 µg/L), the ET-1 content in the serum of rats in the H + low iodine group (16.07 ± 1.67 µg/L) and endothelial injury group (17.38 ± 1.40 µg/L) was higher (P < 0.05). Compared to the control group, the serum levels of vWF in the hyperthyroidism model group (457.75 ± 29.06 ng/L), the H + low iodine group (412.77 ± 28.74 ng/L), the high iodine group (495.36 ± 39.58 ng/L) and the endothelial injury group (402.84 ± 31.61 ng/L) were significantly higher (P < 0.05). The content of P-selectin in the hyperthyroidism model group (4.82 ± 0.81 ng/L), H + low iodine group (4.43 ± 0.64 ng/L) and the endothelial injury group (6.66 ± 0.79 ng/L) was higher than that in the control group (3.77 ± 0.36 ng/L, P < 0.05). Compared with the control group (1.66 ± 0.30 µg/L), the TM content in the serum of the hyperthyroidism model group (2.33 ± 0.51 µg/L) and the endothelial injury group (2.89 ± 0.64 µg/L) was significantly higher, as shown in Table 2.

**Table 2:** Analysis of endothelial injury index in serum of six groups SD rats ($\bar{{\rm{X}}}$ ± S).

Group	NO (µmol/L)	ET-1 (µg/L)	vWF (ng/L)	P-selectin (ng/L)	TM (µg/L)
Control	13.17 ± 2.69	12.99 ± 1.11	376.28 ± 38.23	3.77 ± 0.36	1.66 ± 0.30
Model	25.40 ± 7.72*	16.62 ± 1.40*	457.75 ± 29.06*	4.82 ± 0.81*	2.33 ± 0.51*
H + PTU	16.12 ± 5.00	13.77 ± 1.15	357.76 ± 47.35	3.58 ± 0.52	1.86 ± 0.20
H + low iodine	16.34 ± 4.30	16.07 ± 1.67*	412.77 ± 28.74*	4.43 ± 0.64*	1.75 ± 0.23
High iodine	14.18 ± 4.01	13.14 ± 1.75	495.36 ± 39.58*	4.03 ± 0.54	1.85 ± 0.24
Endothelium injury	32.60 ± 8.90*	17.38 ± 1.40*	402.84 ± 31.61*	6.66 ± 0.79*	2.89 ± 0.64*

*Denotes comparison with control group, P < 0.05. NO, nitric oxide, ET-1, endothelin 1, vWF, von Willebrand Factor, TM, Thrombomodulin.

### Rat descending aorta morphology

**Figure 1:**
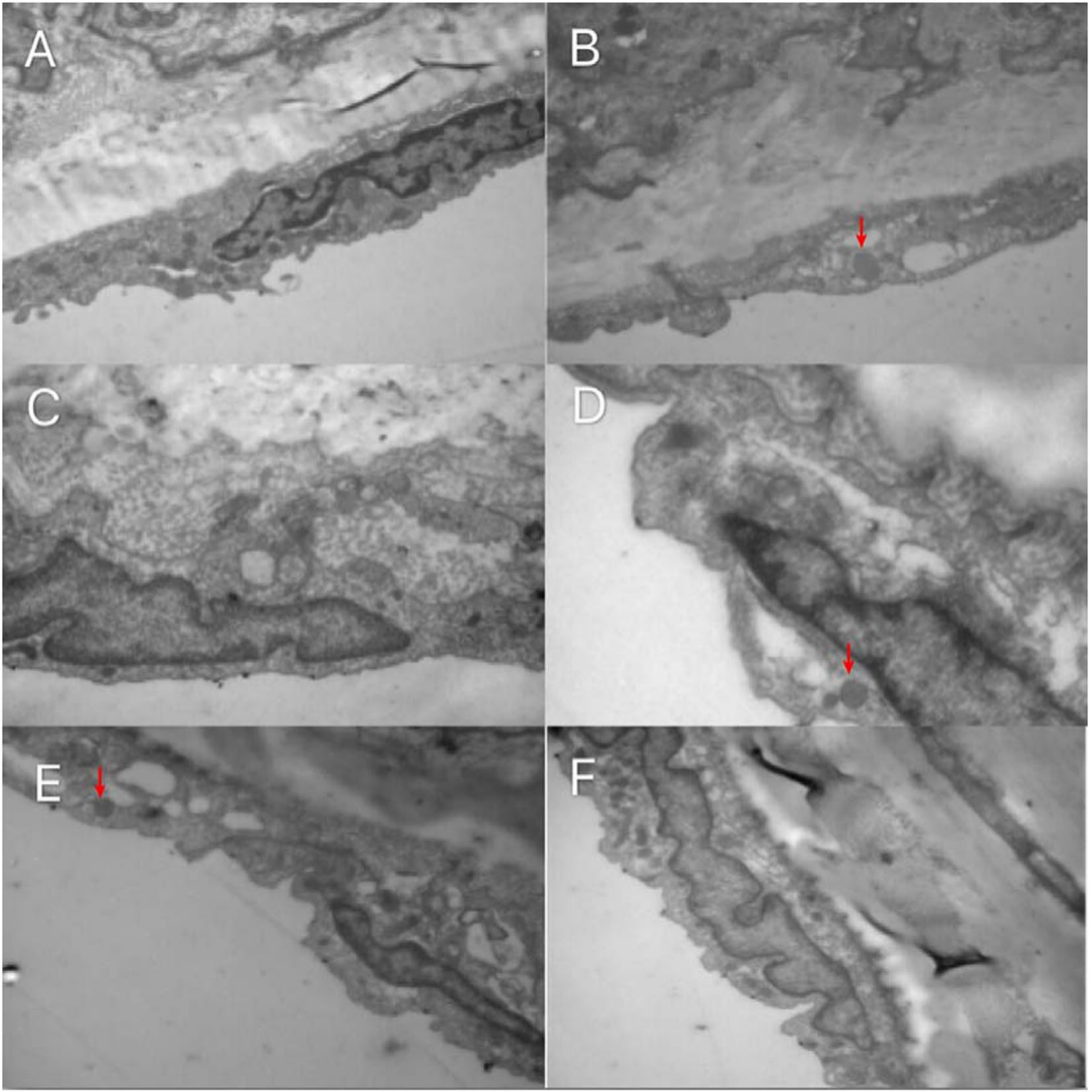
Rat descending aorta morphology. (**A**) control group; (**B**) hyperthyroidism model group; (**C**) hyperthyroidism propylthiouracil group; (**D**) hyperthyroidism low iodine group; (**E**) high iodine group; (**F**) endothelial injury group. In the normal control group, the VEC structure of descending aorta vascular endothelial ultrastructure was intact, endothelial cells and vascular endothelium were closely linked, the basal membrane was intact, the nucleus was spindle shaped, the mitochondria were normal, and the endoplasmic reticulum was regularly arranged. Only mitochondrial enlargement was observed in the hyperthyroidism model group, but no other abnormalities were observed. Partial mitochondrial enlargement was found in the hyperthyroidism low iodine group, but no other abnormalities were observed. There was no significant difference between hyperthyroidism propylthiouracil group and high iodine group and normal control group.Mitochondrial enlargement was obvious in the endothelial injury group, but no other changes were observed.

In the control group, the ultrastructure of vascular endothelial cells in the descending aorta was normal. The endothelial cells were closely linked to the vascular endothelium, the basement membrane was intact, the nucleus was fusiform, the morphology of the mitochondria was normal, and the endoplasmic reticulum was regularly arranged. The hyperthyroidism model group showed only mitochondrial enlargement. In the H + low iodine group, some mitochondria appeared to be swollen. However, no significant differences were observed between the H + PTU group, the high iodine group and the control group. The mitochondria in the endothelial injury group also appeared to be enlarged, as shown in Fig. 1.

### Rat descending aorta EPCs and LOX-1

**Figure 2:**
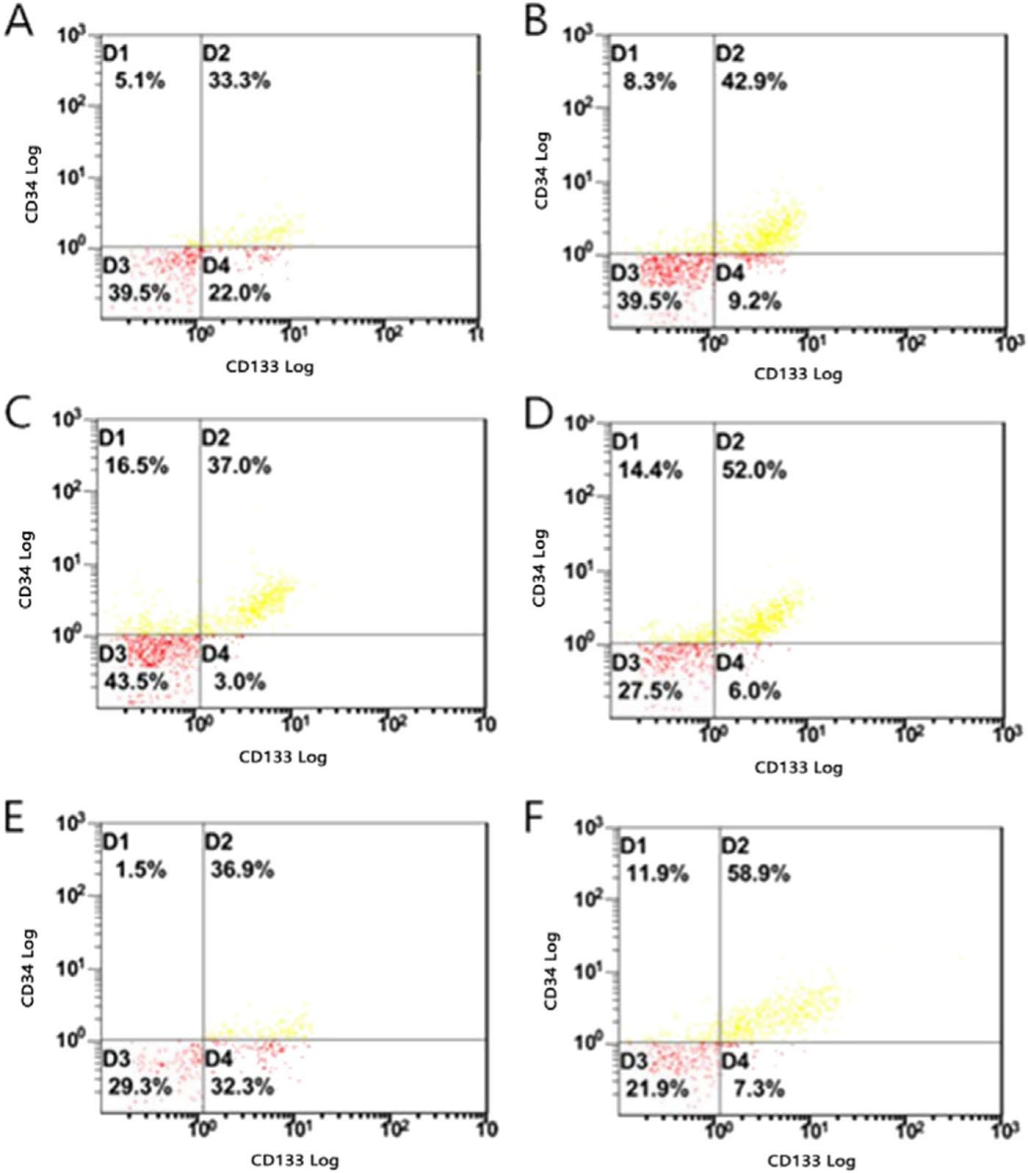
The number of endothelial progenitor cells (EPCs) in descending aorta (determined by flow cytometry). (**A**) control group; (**B**) hyperthyroidism model group; (**C**) hyperthyroidism propylthiouracil group; (**D**) hyperthyroidism low iodine group; (**E**) High iodine group; (**F**)endothelial injury group. Note: The D1 region is a CD34 positive cell population region, the D4 region is a CD133 positive cell population region, and the D2 region is a CD34, and CD133 double positive cell population region (endothelial progenitor cells).

From Table 3 and Fig. 2, the content of EPCs (expressed as a percentage) in the serum of the hyperthyroidism model group, the H + low iodine group and the endothelial injury group were significantly higher than that in the control group.

**Table 3:** Quantitative analysis of EPCs in whole blood of six groups of SD rats.

Group	N	EPCs (%)	N	LOX-1 mRNA (2^−ΔΔCт^)
Control	10	39.04 ± 10.68	11	1.24 ± 1.09
Model	10	51.98 ± 12.77*	11	5.79 ± 7.25*
H + PTU	10	34.22 ± 5.83	11	1.84 ± 0.67
H + low iodine	10	55.78 ± 12.15*	11	5.83 ± 5.06*
High iodine	10	42.42 ± 6.64	11	2.52 ± 1.92
Endothelium Injury	10	61.03 ± 8.57*	11	7.10 ± 4.32*

*Denotes comparison with control group, P < 0.05. EPCs, endothelial progenitor cells.

Additionally, the expression of LOX-1 in the descending aorta of the model group, the H + low iodine group and the endothelial injury group were also significantly higher than that in the control group, as shown in Table 3.

## Discussion

Under physiological conditions, endothelial cells can produce vasoconstriction and vasorelaxation factors to regulate vasomotor responses. Vascular endothelial cells produce NO, an endothelium-derived labile molecule that maintains vascular homeostasis and thus prevents atherosclerotic vascular changes^6^. NO has been shown to inhibit platelet aggregation, leukocyte-endothelium interaction, vascular smooth muscle cell proliferation and migration so that smooth muscle relaxation and arterial dilation can regulate blood pressure and blood flow distribution^7^. ET-1, which is mainly produced by the vascular endothelium, has a strong vasoconstrictive effect and can promote the migration of smooth muscle cells and cause hypertrophy of cardiomyocytes and fibroblast proliferation. By inhibiting prostacyclin synthesis and promoting the release of thromboxane A2, it can aggravate the development of vascular tone and atherosclerosis^8^. The vWF plays an important role in platelet adhesion and aggregation and is mainly synthesized by endothelial cells and megakaryocytes. The vWF promotes platelet aggregation by releasing platelet glycoprotein IB and IIb/IIIa receptor and by releasing multiple platelet granules to aggravate atherosclerosis^9^. Moreover, vWF levels may be a suitable marker to evaluate acute changes in endothelial function because this parameter responds more rapidly to changes in endothelial function than other factors^10^. EPCs are progenitor cells with walking characteristics that can proliferate and differentiate into mature vascular endothelial cells^11^. Recent studies have found that endothelial progenitor cells play an important role in the development of atherosclerosis. When the vascular endothelium is damaged, endothelial progenitor cells can accelerate differentiation and promote vascular repair by homing to the lesion site. Endothelial progenitor cells play an important role in maintaining vascular integrity, repairing vascular endothelial cells and promoting the formation of neovascularization^12^. In this study, the expression of LOX-1 in the descending aorta of the model group, the H + low iodine group and the endothelial injury group was higher than that in the control group. A new type of receptor (lectin-like OX - receptor 1, LOX-1) on human endothelial cells, reported by a foreign study, can absorb oxidized low-density lipoprotein (OX-LDL), referred to as the plant haemagglutinin sample type oxidized low-density lipoprotein receptor 1. The main function is to mediate the uptake of OX-LDL by vascular endothelial cells, and the activation of LOX-1 further induces the production of adhesion molecules, leading to the impairment of endothelial cell function, which is therefore considered one of the important biomarkers of cardiovascular diseases.

### Hyperthyroidism and vascular endothelial injury

Cardiovascular and cerebrovascular diseases are hidden, gradual and systemic, with no obvious clinical symptoms. There are many contributing factors to cardiovascular and cerebrovascular diseases, and endothelial injury is an important feature of cardiovascular and cerebrovascular diseases^13^. The hyperthyroidism model rats have a lower thyrotropin level and have higher thyroid hormones, an enlarged heart, an abnormal heart rate, are irritable and aggressive, and drink and eat more. The results of this experiment showed that the endothelial injury indexes in the serum of the hyperthyroidism group were higher than those in the control group, indicating that the endothelial cells in hyperthyroidism were damaged, which is a high-risk factor for cardiovascular and cerebrovascular diseases. The TSH content may be one of the causes of endothelial cell injury caused by hyperthyroidism. In recent years, the view was held that vascular endothelial cells are also target cells of thyroid hormone^14^, since high thyroid hormone levels lead to endothelial cells producing large amounts of NO, which plays an important role in vasodilation. Ruud S. Kootte *et al*. found that the incidence rate of venous thromboembolism in patients with hyperthyroidism appears to be high in a retrospective cohort study of patients with hyperthyroidism^15^. The results of this study are consistent with these experimental findings that vascular endothelial injury is present in hyperthyroid rats. Studies have confirmed that many endothelial cells synthesize ET and release it into the blood, which contributes to the pathophysiological process of the disease when endothelial cells are damaged. The plasma ET-1 level of hyperthyroidism was significantly higher than that of the normal control group, which might be related to thyroid diseases. The level of plasma ET-1 could be one of the indexes for the judgment of thyroid diseases^16^. The results of the experiment showed that FT_3_ and FT_4_ increased in the hyperthyroidism model group and hyperthyroidism rats with a low iodine diet, and the index of endothelial injury was also higher than that in the control group.

### Propylthiouracil and vascular endothelial injury

Propylthiouracil (hydroxy-2-mercapto-6-propyl pyrimidine; PTU) is a commonly used medicine in the treatment of hyperthyroidism. It acts by inhibiting thyroid peroxidase, thereby blocking the tyrosine iodide and iodine in the thyroid tyrosine condensation and suppressing thyroid hormone synthesis. It also inhibits T_4_ from becoming T_3_ in peripheral tissues, thus resulting in a decline in the more active T_3_ content in the serum. The results from this study show that after an endothelial injury occurs, PTU treatment of hyperthyroidism in rats have effect, because hyperthyroidism can cause endothelial damage, but the use of PTU reduces endothelial damage, as there were no differences between the H + PTU group and the control group. These results could mean that PTU not only affects hyperthyroidism in rats but may also prevent the occurrence of endothelial injury. PTU can induce ANCA-associated vasculitis^17^. After treatment, hyperthyroid rats showed a normal heart rate, metabolism and other symptoms, and vascular endothelial injury was greatly reduced. Interestingly, considering the heart-body ratio, no difference was found between the PTU group and the hyperthyroidism model group, likely because there are indeed some indicators have a higher distribution, which may influenced the statistic results and myocardial hypertrophy symptoms cannot be repaired in a short time period.

### Iodine and vascular endothelial injury

Iodine is one of the essential trace elements in the human body. Globally, iodine deficiency disorders are effectively controlled by salt iodization. At the same time, the problem of excessive iodine in areas with high contents of iodine in water has attracted increasing attention^18,19^. Related studies have shown that high iodine has multiple effects, such that it can promote and inhibit the proliferation of endothelial cells, i.e., an appropriately high amount of iodine can promote endothelial cell proliferation, but excess iodine inhibits cell proliferation. The differences in its effects are related to its concentration and duration^20^. Cell experiments have shown that high amounts of iodine cause endothelial cell damage by inhibiting cell proliferation, destroying a cell’s antioxidant capacity, and promoting the production of NO and adhesion factor expression^5^. High levels of iodine cause a gradual increase in many active plasma factors in rats and activate endothelial cells. Malnutrition can also aggravate the effects of excessive iodine by leading to the activation of the inferior vena cava endothelial cells in rats^4^. Conversely, in the absence of iodine, the activation of the vascular endothelium will be lower, thus affecting vascular endothelial function.

The results of this study showed that for SD rats with an intake of 3,000 µg/L of iodine water, the endothelial injury index was not all abnormal; only the vWF was higher than that in the control group, which may be related to the length of feeding time. High levels of iodine do not directly cause injury to endothelial cells in healthy rats, contrary to the results of some previously published papers.

This experiment simulated the effects and treatment of hyperthyroidism by feeding a low-iodine diet and administering propylthiouracil in the treatment of hyperthyroidism. This study aims to observe the effect of these treatments on rat vascular endothelial cells. The results showed that hyperthyroidism could lead to endothelial cell damage, which may be a high-risk factor for cardiovascular and cerebrovascular diseases. In this study, PTU not only exhibited a therapeutic effect on the hyperthyroid rats but also prevented the occurrence of endothelial injury. In most articles, high levels of iodine can lead to the proliferation and damage of endothelial cells; however, most of these articles were cell experiments, rather than animal experiments. The results of animal experiments have shown that when SD rats’ drinking water contained 3000 μg/L iodine, no abnormal endothelial injury indicators were observed, which may be related to the length of feeding time. Therefore, the effect of high levels of iodine on endothelial injury in healthy rats was not significant in this study.

The results show that high levels of iodine do not directly cause injury to endothelial cells in healthy rats, which is contrary to the results of some previously published papers. In this study, animal models were utilized, while others have mostly used cell lines to study the effects of iodine on endothelial cell proliferation and injury.

## Conclusions

Hyperthyroidism can damage vascular endothelial cells and is a high-risk factor for cardio-cerebrovascular disease. Propylthiouracil could be used in the treatment of hyperthyroidism, thus protecting endothelial cells from damage. The effect of high iodine on vascular endothelial injury in rats is not definite, and further verification is needed.

## Data Availability

Data were not open available on line. Data might be offered on special requirement if applied.
